# A questionnaire study comparing the attitudes of adolescents and young adults (AYA) and older adult cancer patients towards early phase clinical trials

**DOI:** 10.1186/s13063-025-09069-1

**Published:** 2025-11-11

**Authors:** Eleanor Johnston, Martin McCabe, Louise Carter

**Affiliations:** 1 Division of Cancer Sciences, Faculty of Biology, Medicine and Health, University of Manchester, Manchester, UK; 2 The Christie NHS Foundation Trust, Manchester, UK

**Keywords:** Adolescent and young adults, Cancer, Clinical trials, Attitudes, Acceptability

## Abstract

**Background:**

Adolescent and young adults (AYA) with cancer are poorly recruited to early phase clinical trials compared to older adult patients, contributing to a slower improvement in survival outcomes. To overcome this, we need to better understand the barriers to recruitment. This study aimed to explore the attitudes of AYA with cancer (aged 16–24) towards clinical trials, compared with those of older patients (aged 25 and over).

**Methods:**

This questionnaire-based study was conducted in a regional cancer centre in North-West England. Participants were eligible if they were previously diagnosed with cancer, and either in follow up, receiving or being considered for treatment. Responses were collected online using the Qualtrics™ tool. The outcome measure was the Cancer Treatment sub-scale of the Attitudes Toward Cancer Trials Scales (ACTS-CT), and nine additional questions as piloted for content and validity by Lewin et al. (2020).

**Results:**

Fifty AYA [64% female] and 51 non-AYA [43% female] completed at least part of the questionnaire. At both participation and at initial diagnosis, most AYA respondents were aged 19–24 [62% and 50% respectively], while most non-AYA respondents were 30 or over [88%]. The most common diagnosis in AYA was lymphoma [46%], while in non-AYA it was carcinoma [43%]. Significant differences were observed between AYA and non-AYA participants in the personal benefits (*p* = 0.000) and personal and social value (*p* = 0.001) categories, and four of the additional statements including the importance of who approaches the patient (*p* = 0.000), concerns about the impact of trials on short term (*p* = 0.005) and long-term goals (*p* = 0.000), and feeling too overwhelmed to participate in a trial (*p* = 0.000). No significant differences were found in the personal barriers and safety and trust in the research process categories.

**Conclusions:**

Understanding the attitudes and concerns of AYA patients is crucial for the development of patient-centered approaches to clinical trial participation in this population. Here, we identified notable differences in the perspectives of AYA versus older patients that can be used to address inequalities in this group.

**Supplementary Information:**

The online version contains supplementary material available at 10.1186/s13063-025-09069-1.

## Introduction

Adolescents and young adults (AYA) with cancer, defined as patients aged 16–24 years, are a unique patient group with unmet medical needs. Cancer in AYA is rare and accounts for less than 1% of all cancer cases in the United Kingdom (UK) [1], but differs to older adults and young children (non-AYA) not only in the common cancers that affect them, but also their needs and priorities [2–4]. While some cancer types, such as leukemias and lymphomas, have seen improvements in survival outcomes among AYA [5, 6], improvements have not been observed at the same rate as in non-AYA [3, 5, 7, 8]. There are still cancers, including bone and soft tissue sarcomas (STS) and brain tumours, that continue to have unacceptably poor prognosis rates among AYA [5, 7]. To improve outcomes for AYA, new treatments are urgently needed [2, 3].

Explanations for the slow progress in improving AYA outcomes include but are not limited to, differences in the biology of AYA cancer compared to non-AYA [9], scarcity in advancements in the availability of new treatments and lack of suitable trials for this age group, and poor recruitment to clinical trials [10–14]. Prior literature has shown AYA recruitment to trials to be poor [3, 15–18] compared to paediatric patients. This under-representation of AYA in trials is an international problem and similar barriers exist irrespective of location or model of healthcare. Poor enrolment in clinical trials and poor availability of trials for this age group, exacerbated by eligibility criteria that exclude patients on the basis of age rather than biology or pharmacokinetics, delays testing of new drugs in AYA patients.

Early phase cancer clinical trials (EPCTs, phase I/II) are designed to investigate the safety of a new drug, or combination of drugs, and look for early signals of anti-tumour activity [8, 19]. As we move into an era of precision medicine, many novel cancer drugs are only available through clinical trials and are therefore inaccessible to those not participating in trials [20].

Explicit, nationally coordinated efforts to improve trial participation rates for children and young people in the UK have been ongoing since 2005 [21], with the most recent National Health Service (NHS) Long-Term Plan pledging to support and increase AYA participation in trials to 50% by 2025 [22].

In 2014, Fern et al. in the UK demonstrated five factors impacting trial participation for AYA with cancer: availability, accessibility, awareness, appropriateness and acceptability [4]. In the context of EPCTs, availability is largely reliant on the development and testing of new drugs relevant to the cancers typical of adolescence and young adulthood [4], many of which are rare cancers [14]. Open trials should be accessible to all eligible AYAs, irrespective of where the patient lives or the location and age specialisation of their primary treatment in a paediatric or adult hospital. Another reported barrier to AYA EPCT access is professional gate-keeping [23]. EPCT awareness involves treating oncologists having knowledge of appropriate trials for their AYA patients and being proactive in presenting trial options to their patients [2, 4, 24]. Appropriateness largely relates to eligibility criteria, which may explicitly exclude AYA patients because of arbitrarily defined upper and lower age limits or inadvertently exclude them because of the age criteria for treatment in the centres where specific trials are open, which are typically either adult- or child-focused, resulting in some AYA being considered too old or too young to be recruited [3, 25]. Multiple studies have identified a gap in access to experimental interventions for patients younger than 18, summarised by a meta-research analysis, which demonstrated that patients aged 18 years had access to 2.8 times more trials investigating chemotherapies than patients aged 17 years, 4.6 times more trials of molecular targeted therapies, and 12 times more immunotherapy trials [17]. Lastly, EPCT acceptability to AYA patients can be affected by trial design, assessment timepoints, methods of information sharing and recruitment and AYA attitudes toward clinical trials [18, 26], which may differ compared to the attitudes of other age groups [27]. Improving AYA trial enrolment will require collaboration among different stakeholders, and a key aspect to consider will be the acceptability of clinical trials to AYA patients, and their likelihood of participating, if offered. To investigate this, we set out to explore the attitudes of adolescent and young adult (AYA) cancer patients towards clinical trials and compare these with the attitudes of older adult cancer patients.

## Methods

### Participants, eligibility criteria and sample size estimate

Participants were recruited simultaneously into AYA and non-AYA cohorts.

The inclusion criteria for this study were patients (1) previously diagnosed with cancer at any age, and either in follow up at any time point after treatment, receiving active treatment or being considered for treatment; (2) aged between 16 and 24 years at the time of participation for the AYA cohort, or 25 years and over for the non-AYA cohort. Participants were excluded from the study if they were serving a custodial sentence or incapable of completing the survey, for example due to conscious level, mental incapacity or being unable to understand English.

### Study design

This was a single-site questionnaire-based study conducted at The Christie NHS Foundation Trust in Manchester, UK. All questionnaire responses were collected online, using the Qualtrics™ tool. The survey is reported here in accordance with the Checklist for Reporting Of Survey Studies (CROSS, Supplementary file 1). Participants were cancer patients identified and recruited across two teams: the AYA service and the Experimental Cancer Medicine Team (ECMT). The former provides care for cancer patients aged 16 to 24 years, and the latter is an EPCT team that offers first-in-human and first in-combination trials to cancer patients aged 16 and over.

Inpatients and outpatients attending the AYA service and the ECMT clinics were signposted by their healthcare professionals to the study advertisement which had a QR code linking to the patient information sheet (PIS) on Qualtrics™. In addition, for recruitment to the AYA cohort, a member of the AYA multidisciplinary team (MDT) identified all AYA patients who attended appointments at the Christie Hospital during the calendar years 2020 and 2021 and sent a text message to approximately 90 eligible participants advertising the study. The study was also advertised by a post on the AYA service’s Facebook page. The study was approved by the London Bridge Research Ethics Committee (reference: 22/PR/1199) and the study opened to recruitment on 03/10/2022.

### Data collection

The outcome measure was the Cancer Treatment sub-scale of the Attitudes Toward Cancer Trials Scales (ACTS-CT) [28]. This questionnaire consists of statements to assess patient attitudes towards cancer clinical trials, comprising 18 questions over four categories: personal benefits; personal barriers and safety; personal and social value; and trust in the research process [28]. The present study also included nine additional questions which were piloted for content and validity in a sample of AYAs by Lewin et al., in their 2020 paper [29]. Informed consent was obtained from all participants included in the study. In the patient information sheet, we defined early phase clinical trials and explained the purpose of the study was to explore participant attitudes towards them (Supplementary file 2). All participants were presented with the same survey items, which included demographic questions, items from the ACTS-CT scale and the additional questions validated by Lewin and colleagues. Respondents rated all 27 questions with a 7-point Likert-type rating scale (ranging from 1 = strongly disagree to 7 = strongly agree). Inverted scales were used for questions that were worded negatively compared to other items in the category as validated by Lewin et al. Participants were also asked if they had previously relapsed and if they had previously participated in a clinical trial. The answers were categorical: participants chose between a predefined list of possible answers as shown in supplementary file 3.

### Statistical analysis

Data from the questionnaire responses were analysed in RStudio [30]. Results from those patients who did not complete the demographic questions were excluded from analysis. Descriptive statistics were used to describe the demographics of the respondents. Means and standard deviations were calculated for each of the 27 questions across both the four categories in the ACTS-CT and the additional questions. For each participant, a total score was calculated for each category in the ACTS-CT. A total score was not included in the analysis for a category if respondents left at least one question in a category blank. Additionally, total scores were calculated for every participant for both ACTS-CT and ACTS-CT plus additional questions.

Mann–Whitney *U* tests were used to compare differences between total scores of the AYA and non-AYA cohorts for each of the four categories and each of the nine additional questions separately. A *p*-value of < 0.05 was considered statistically significant.

## Results

### Baseline characteristics

A total of 92 participants (44 AYA and 48 non-AYA) completed the questionnaire in full and 9 participants (6 AYA and 3 non-AYA) completed at least part of the questionnaire. A further 7 participants did not complete the demographic questions and were excluded from analysis. Demographic information for the participants is presented in Table 1.

**Table 1:** Demographics

**Characteristic**	**AYA *N*** (%)	**Non-AYA *N*** (%)
***N***	50	51
***Age at diagnosis***		
15 or younger	8 (16)	3 (6)
16–18	17 (34)	0
19–24	25 (50)	4 (8)
25–29	-	2 (4)
30 or over	-	42 (82)
***Age at participation***		
16–18	19 (38)	-
19–24	31 (62)	-
25–29	-	6 (12)
30 and over	-	45 (88)
***Diagnosis***		
Leukaemia	5 (10)	1 (2)
Lymphoma	23 (46)	4 (8)
Brain or spinal cord tumour	5 (10)	2 (4)
Bone or soft tissue sarcoma	8 (16)	2 (4)
Germ cell tumour	2 (4)	3 (6)
Carcinoma	3 (6)	22 (43)
Other/not sure	4 (8)	17 (33)
***Cancer has previously relapsed***		
Yes	12 (24)	37 (73)
No	38 (76)	14 (27)
***Gender***		
Male	18 (36)	29 (57)
Female	32 (64)	22 (43)
***Ethnicity***		
Asian	7 (14)	1 (2)
White	37 (74)	49 (96)
Other	6 (12)	1 (2)
***Previous trial participation***		
Yes	9 (18)	32 (63)
No	36 (72)	18 (35)
Not sure	5 (10)	1 (2)

Most AYA respondents were aged 19–24 at both diagnosis and participation (50% and 62%, respectively) and most non-AYA respondents were 30 or over at diagnosis and participation (82% and 88%). The most common AYA diagnoses were lymphoma (46%), bone or STS (16%) or brain or spinal cord tumour (16%), whilst the most common non-AYA diagnoses were carcinomas (43%) or ‘other/not sure’ (33%). Most AYA had not relapsed (76%), whereas most non-AYA had relapsed (73%). Of participants who could remember whether they had previously been involved in a clinical trial, 20% of AYA had participated in trials compared to 64% of non-AYA.

### Comparison of AYA vs non-AYA

Results for all questions are displayed in Table 2. Significant differences were observed between AYA and non-AYA participants in the categories of personal benefits and personal and social value. However, no significant differences were found in the categories of personal barriers and safety and trust in the research process.

**Table 2:** Full set of results

Statement	TYA mean (± SD)	Non-TYA mean (± SD)	Difference between means (TYA minus non-TYA)	*p*-value
Personal benefits	**15.06** (**± 4.28**)	**19.62** (**± 4.72**)	**− 4.56**	**0.000**
Q1. I’d get improved cancer treatment if I took part in a cancer study	4.02 (± 1.33)	4.82 (± 1.72)	− 0.8	
Q2. People who join cancer studies have a better chance of beating their cancer	3.76 (± 1.39)	4.92 (± 1.51)	− 1.16	
Q3. Joining a cancer study would mean I’d receive the best existing cancer treatment	3.70 (± 1.31)	5.25 (± 1.29)	− 1.55	
Q4. By joining a cancer study, I would receive better health care	3.58 (± 1.39)	4.66 (± 1.49)	− 1.08	
Personal barriers and safety	**22.79** (**± 4.34**)	**22.42** (± **5.16**)	**0.37**	**0.758**
Q5. Taking part in a cancer study is a lot more trouble than just getting the usual treatment*	4.92 (± 1.47)	4.34 (± 1.62)	0.58	
Q6. Getting treatment in a cancer study is less safe than getting the usual treatment*	4.51 (± 1.17)	4.72 (± 1.49)	− 0.21	
Q7. Treatments received in a cancer study could be unsafe for myself*	4.12 (± 1.56)	4.16 (± 1.50)	− 0.04	
Q8. My taking part in a cancer study could lead to more health problems*	4.20 (± 1.27)	3.86 (± 1.32)	0.34	
Q9. Joining a cancer study would make cancer treatment more difficult*	4.96 (± 1.22)	5.34 (± 1.10)	− 0.38	
Personal and social value	**28.64** (**± 4.89**)	**31.65** (**± 3.28**)	− **3.01**	**0.001**
Q10. In general, people should know more about cancer studies	5.92 (± 1.45)	6.16 (± 0.98)	− 0.24	
Q11. Cancer studies are of little importance to me*	5.49 (± 1.61)	6.22 (± 1.23)	− 0.73	
Q12. Access to cancer treatment studies is important to me	5.19 (± 1.33)	6.22 (± 0.94)	− 1.03	
Q13. People who take part in cancer studies are helping all of us fight cancer	6.11 (± 1.22)	6.53 (± 0.77)	− 0.42	
Q14. I feel certain my safety would be watched closely in a cancer study	5.89 (± 1.20)	6.51 (± 0.82)	− 0.62	
Trust in the research process	**22.59** (**3.65**)	**23.78** (**3.73**)	**− 1.19**	**0.079**
Q15. Doctors and nurses tell patients the truth about what to expect during a cancer study	5.98 (± 1.22)	6.35 (± 0.90)	− 0.37	
Q16. If I took part in a cancer study, I would be treated like a guinea pig*	5.72 (± 1.33)	5.41 (± 1.55)	0.32	
Q17. Doctors and nurses mislead their patients who are involved in cancer studies*	5.85 (± 1.10)	6.33 (± 1.03)	− 0.48	
Q18. It would be safe for me to join a cancer study for treatment	4.93 (± 1.34)	5.69 (± 1.19)	− 0.76	
Additional questions				
Q19. I would be more likely to participate in a clinical trial if my current cancer treatment was not effective or available	5.37 (± 1.55)	5.86 (± 1.37)	− 0.49	0.088
Q20. The greater the severity of my illness/cancer, the more likely I would be willing to participate in a clinical trial	5.35 (± 1.52)	5.57 (± 1.73)	− 0.22	0.265
Q21. It doesn’t matter who approaches me about a clinical trial as long as they have all of the necessary information to answer any questions that I have	3.96 (± 1.94)	5.37 (± 1.60)	− 1.41	0.000
Q22. I am worried about how a clinical trial can affect my daily life or short-term goals (work and school). *	3.54 (± 1.64)	4.51 (± 1.58)	− 0.97	0.005
Q23. I am worried about how a clinical trial can affect my long-term goals (starting a family, career/other goals). *	3.18 (± 1.57)	4.65 (± 1.65)	− 1.48	0.000
Q24. After being approached for a clinical trial, I would seek additional information from the internet. *	3.62 (± 1.76)	4.02 (± 1.92)	− 0.40	0.289
Q25. My friends’ and family’s opinions matter to me when making a decision about clinical trials	4.91 (± 1.81)	4.78 (± 1.75)	0.13	0.660
Q26. I am too overwhelmed to consider participation in a clinical trial. *	4.70 (± 1.37)	5.92 (± 1.26)	− 1.22	0.000
Q27. I would consider my ability to meet the demands of the trial protocol (i.e., frequency of visits, procedures, travel time)	5.30 (± 1.32)	4.63 (± 1.78)	0.67	0.084
Total ACTS-CT	**89.82** (**± 9.67**)	**97.29** (**± 11.90**)	**− 7.67**	**0.001**
Total questionnaire	**129.75** (**± 13.30**)	**142.44** (**± 14.96**)	**− 12.69**	**0.000**

^*^Likert scale scores for these questions have been reversed for analysis

Data with bolded emphasis highlight category and overall totals

#### Personal benefits

Total scores for 100 participants were available for analysis of the personal benefits category (AYA, 50, 100%; non-AYA, 50, 98%). There was a difference between the scores of AYA and non-AYA patients when considering perceived personal benefits of trial participation, with total scores for this category resulting in a AYA mean of 15.06 (SD 4.28) and a non-AYA mean of 19.62 (SD 4.72). This suggests that on average, AYA patients were less likely than non-AYA to believe they would personally benefit with regards to their treatment and healthcare from taking part in a clinical trial.

#### Personal barriers and safety

Total scores for 97 participants were available for this analysis (AYA, 47, 94%; non-AYA, 50, 98%). There was not a significant difference (*p* = 0.758) between the scores of AYA and non-AYA patients for this category or the individual items when considering perceived personal safety and barriers toward trial participation, with total scores resulting in a AYA mean of 22.79 (SD 4.34) and a non-AYA mean of 22.42 (SD 5.16). This indicates that AYA participants were no less likely than non-AYA to believe they would experience safety risks or difficulties if taking part in a clinical trial.

#### Personal and social value

Total scores for 96 participants were available for this analysis (AYA, 47, 94%; non-AYA, 49, 96%). There was a statistically significant difference (*p* = 0.001) between the scores of AYA and non-AYA patients when considering the perceived value of trial participation: category sums resulted in a AYA mean of 28.64 (SD 4.89) and non-AYA mean of 31.65 (3.28). This suggests that AYA patients were less likely than non-AYA to believe clinical trials have value for the population and personally.

#### Trust in the research process

Total scores for 95 participants were available for this analysis (AYA, 46, 92%; non-AYA, 49, 96%). There was not statistically significant difference (*p* = 0.079) between the scores of AYA and non-AYA patients, with total category scored resulting in a AYA mean of 22.59 (SD 3.65) and non-AYA mean of 23.78 (SD 3.73) when considering trust in the research process. This suggests that there was no major difference between the views on AYA and Non-AYA participants with regards to the research process.

#### Additional questions

Significant differences were identified between AYA and non-AYA patients for four statements, including the importance of who approaches the patient (Q21, *p* = 0.000), concerns about the impact of trials on short term goals (Q22, *p* = 0.005) long-term goals (Q23, *p* = 0.000) and being too overwhelmed to participate in a trial (Q26, *p* = 0.000).

## Discussion

This pivotal study is the first to explore the attitudes of AYA cancer patients towards taking part in clinical trials and compare these with the attitudes of older adult cancer patients in an early phase trial context. The findings form the first analysis using this questionnaire in the UK, addressing the unmet need for validated questionnaires specifically designed to explore attitudes to EPCTs.

There are no validated questionnaires specifically designed to explore attitudes to EPCTs. We chose to use this validated questionnaire because it had face value, demonstrated by the relevance of the questionnaire contents when compared to our shared clinical experience of the fears and concerns expressed by young and older adult patients referred for EPCTs.

Significant findings for the personal benefits and personal and social value categories indicate that AYA participants were less inclined to believe they would receive personal benefits from clinical trial participation compared to non-AYA participants, and also that AYA patients were less likely to see the value of clinical trials both for themselves and the cancer community. It is worth noting that although non-AYA scored more highly than AYA participants, both groups overall agreed with the statements in this category.

In contrast, there were no notable differences between AYA and non-AYA with regards to their views on trial safety, and both groups disagreed with the statements on average. Additionally, there was no statistically significant difference between the scores of AYA and non-AYA for the trust in research category, suggesting that AYA patients were no less likely than non-AYA to trust clinical trials and the professionals involved in research.

For the additional questions, significant differences were identified between AYA and non-AYA patients for four of the nine statements. These were concerns about the impact of trials on short- and long-term goals, the importance of who approached them about a clinical trial and being too overwhelmed to participate in a trial.

One explanation for the differing importance of who approaches them could be explained by differences in the approach to AYA versus adult care, as the former are often treated by smaller groups of professionals, all have a named AYA specialist nurse and therefore may build stronger relationships with their team. However, it is important to consider potential bias in the sample due to the recruitment method of respondents through a specialised AYA service, which may not equally reflect AYA being treated in non-AYA settings.

Consistent with prior research, there was a trend toward AYAs being worried about how a clinical trial can affect their daily life or short-term goals (i.e. work and school) (*p* = 0.005). Sodergren et al. found differences in relevant and important health-related quality of life (HRQoL) concerns between AYA and non-AYA cancer patients with different priorities for AYA cancer patients (aged 14–25 years) to those of older adult patients (aged 26–60 years) with cancer. When all participants scored the relevance and importance of areas of HRQoL concern, several were recognised as relevant and important across all age groups including symptoms, impact on hobbies and leisure time activities, and emotional impact. However, they found issues that were more relevant or important to AYAs compared with non-AYAs included; interruption to education and greater motivation to succeed academically [27]. It is clear that AYA have distinct needs and values of that differ to non-AYA, and it therefore essential that we are able to tailor engagement strategies specifically for them in order to improve recruitment.

A previous study conducted by Lewin et al., using the same set questionnaire, compared the attitudes of AYAs (aged 15–39) with non-AYAs (aged 40 and over) with a cancer diagnosis towards clinical trial participation in a Canadian cancer centre. Our significant results in the personal benefits and personal and social value categories contrasts their finding of no significant differences between AYA and non-AYA participants for both categories. In their study, Lewin et al. found that the difference between AYA and non-AYA was not statistically significant for the trust in the research category, with a p-values of 0.93 [29]. Additionally, for the ‘Personal barriers and safety’ category, we did not find a significant difference, but Lewin et al. found in their comparison for the same category was statistically significant (*p* = 0.01). Among the additional questions, we found significant differences for four statements (Q21, Q22, Q23 and Q26). In their paper, Lewin et al. found a statistically significant difference between AYA and non-AYA for only one of the statements, with AYAs being more worried regarding the effect of trial participation on their long-term goals (Q23) (*p* = 0.04). Comparing our results with the study by Lewin et al. is difficult, due our differing definitions of the TYA/AYA age bracket. While the definition and consensus of AYA varies in the literature, we used 16–24, in line with NHS guidance in the UK and the demographic treated by the Christie AYA team.

This study has limitations that should be considered when interpreting the findings. Firstly, the self-selecting nature of the participants. The majority (63%) of non-AYA participants were already enrolled on trials, and the remainder were predominantly recruited through ECMT because they were being considered for EPCTs, although not all non-AYA respondents had experience of trials at the time of participation All AYA participants were recruited regardless of their trial status. This limits the generalisability of the findings. Additionally, among our respondents, the proportions of patients who had relapsed in each group differed, in that most AYA had not relapsed, while most non-AYA had relapsed. This is likely to influence individual patient approaches to considering the risk versus benefits of enrolling in trials beyond their age alone.

The study also lacked diversity in terms of ethnicity, with majority (74% of AYA, 96% of non-AYA) self-reporting as white. Future analyses should involve comparison of responses and between different ethnic groups, cancer types and sex. It would also be useful to compare between cancer types and exploring differences for patients with cancer types for which there are generally fewer trials available, such as brain tumours.

Finally, while the ACTS-CT data collection tool encompasses several important and relevant topics, it is not specific to EPCTs trials which may limit the ability to draw conclusions specifically related to EPCT attitudes. In our study, we used the patient information sheet to introduce the questions and frame them around early phase trial recruitment; however, the questions themselves do not specifically address this. The development of a validated tool specifically designed for exploring attitudes towards early phase trials would be beneficial for future research in this area. There remains a need for further research with larger sample sizes and more diverse populations to validate and expand upon these findings.

Understanding the attitudes and concerns of AYA patients is crucial for the development of patient-centred approaches to clinical trial recruitment and participation in this population. Overall, the findings of this study contribute to the existing literature on the barriers for AYA trial participation and further the evidence base around the acceptability of trials for AYA. To our knowledge, this is the first analysis of this questionnaire in a UK population and the differences we identified in perspectives across the questionnaire present opportunity for the provision of specialist care to help to support AYA with their specific concerns.

## Supplementary Information

Additional file 1. CROSS survey checklist.

Additional file 2. Patient information sheet

Additional file 3. Questionnaire

## Data Availability

The datasets used and/or analysed during the current study are available from the corresponding author on reasonable request.

## Abbreviations

ACTS-CT: Attitudes Toward Cancer Trials Scales–Cancer Treatment
AYA: Adolescent and Young Adult
ECMT: Experimental Cancer Medicine Team
EPCT: Early Phase Clinical Trials
MDT: Multi-Disciplinary Team
NHS: National Health Service
PIS: Patient Information Sheet
SD: Standard Deviation
STS: Soft Tissue Sarcoma
UK: United Kingdom
