# Supplementary material for: A questionnaire study comparing the attitudes of adolescents and young adults (AYA) and older adult cancer patients towards early phase clinical trials
Source: Trials. 2025 Nov 11;26:488. doi: 10.1186/s13063-025-09069-1 (PMC12606790; doi:10.1186/s13063-025-09069-1)
Supplement: Supplementary file 2 — Additional file 2. Patient information sheet [file 13063_2025_9069_MOESM2_ESM.docx]

**Exploring the attitudes of teenager and young adults with cancer towards early phase clinical trials**

You are being invited to take part in a research study exploring the attitudes of teenage and young adult (TYA) cancer patients (aged 16-24) towards early phase clinical trials and comparing these with the attitudes of non-TYA cancer patients (25 and over). This research is being done as part of a master’s degree. Before you decide whether to take part, it is important for you to understand why the research is being conducted and what it will involve. Please take time to read the following information carefully before deciding whether to take part and discuss it with others if you wish.

**About the research**

**Who will conduct the research?**

The Chief Investigator for this study is Dr Louise Carter, Clinical Senior Lecturer in the Division of Cancer Sciences, University of Manchester. Co-Investigators include Dr Martin McCabe, Clinical Senior Lecturer in Paediatric and Adolescent Oncology at the University of Manchester and Eleanor Johnston, MRes Student, University of Manchester.

**What is the purpose of the research?**

Early phase clinical trials are the first step in getting new cancer treatments to patients. We know that not enough teenagers and young adults (TYA, aged 16-24) with cancer enrol in clinical trials, so we want to better understand their attitudes towards them. We would also like to compare TYA attitudes with older adult cancer patients (25 and over) being treated within the Experimental Cancer Medicine Team (ECMT). We hope this study will help us to further understand the barriers to involving TYA in early phase trials.

**Am I suitable to take part?**

You are eligible to participate in this research if you are aged 16-24 and either on treatment or under follow up from the TYA service at the Christie, or if you are aged 25 or over and are being reviewed by the ECMT at the Christie.

**Will the outcomes of the research be published?**

As this study forms the basis of an academic project to obtain a qualification, the outcomes of this study will be reported as a project report to the academic supervisors assigned to the student obtaining the qualification. Furthermore, findings may be disseminated in an appropriate peer reviewed scientific journal and presented at relevant conferences.

**What would my involvement be?**

**What would I be asked to do if I took part?**

For this study, we will ask you to complete a single questionnaire. This will take approximately 10-15 minutes to complete.

If you experience any issues or feel distressed at any time whilst completing this questionnaire, please contact The Christie Patient Advice and Liaison Service (PALS) at 0161 446 8217 or on the-christie.pals@nhs.net.

**Will I be compensated for taking part?**

You will not be paid for taking part, but if you chose, you can be entered into a prize draw for the chance to win a £50 gift voucher.

If you would like to enter the prize draw, you can enter your email address following completion of the questionnaire. This information cannot be used to contact you regarding any other matter, and it will not be used to make decisions about future services available to you. Please note email addresses will be stored separately from the anonymous questionnaire data.

**What happens if I do not want to take part or if I change my mind?**

It is up to you to decide whether or not to take part. If you do decide to take part your consent will be implied by completing the questionnaire. It will not be possible to remove your data from the project once it has been submitted as it is entirely anonymous, and we will not be able to identify your specific data. However, if you provide an email address for the prize draw, this will be considered confidential information and you are able to withdraw this at any time by emailing either Dr Carter (louise.carter@manchester.ac.uk) or Dr McCabe (Martin.McCabe@manchester.ac.uk) or by speaking to the medical team within either the TYA unit or ECMT.

**Data Protection and Confidentiality**

**What information will you collect about me?**

Completion of the questionnaire is anonymous, and we will not need to collect information that could identify you. The only personal information we will collect is your email address if you choose to participate in the prize draw.

**Will my participation in the study be confidential and my personal identifiable information be protected?**

All email addresses will be stored in a secure electronic file that will be separate from the questionnaire data. All data will be stored on a secure password protected server at the Christie NHS Foundation Trust. Data will be accessed by members of the research team only and will not be stored on the hard drives of laptops or computers used by the research team. Electronic files will be encrypted with their own unique password.

**What if I have a complaint?**

If you have a complaint that you wish to direct to members of the research team, please contact Dr Carter or Dr McCabe (louise.carter@manchester.ac.uk or Martin.McCabe@manchester.ac.uk).

**If you wish to make a formal complaint to someone independent of the research team or if you are not satisfied with the response you have gained from the researchers in the first instance, then please contact:**

The Research Ethics Manager, Research Office, Christie Building, The University of Manchester, Oxford Road, Manchester, M13 9PL, by emailing: research.complaints@manchester.ac.uk or by telephoning 0161 306 8089.

If you wish to contact us about your data protection rights, please email dataprotection@manchester.ac.uk or write to The Information Governance Office, Christie Building, The University of Manchester, Oxford Road, M13 9PL at the University and we will guide you through the process of exercising your rights.

You also have a right to complain to the Information Commissioner’s Office about complaints relating to your personal identifiable information Tel 0303 123 1113

You can also contact The Christie Patient Advice and Liaison Service (PALS):

Tel: 0161 446 8217

Email: the-christie.pals@nhs.net.
