# Supplementary material for: A questionnaire study comparing the attitudes of adolescents and young adults (AYA) and older adult cancer patients towards early phase clinical trials
Source: Trials. 2025 Nov 11;26:488. doi: 10.1186/s13063-025-09069-1 (PMC12606790; doi:10.1186/s13063-025-09069-1)
Supplement: Supplementary file 3 — Additional file 3. Questionnaire [file 13063_2025_9069_MOESM3_ESM.pdf]

Start of Block: Demographics questions

Q1 How old were you when you were first diagnosed with cancer?

- ○ 15 or younger (1)
- ○ 16-18 (2)
- ○ 19-24 (3)
- ○ 25-29 (4)
- ○ 30 or over (5)

Q2 How old are you now?

- ○ 16-18 (1)
- ○ 19-24 (2)
- ○ 25-29 (3)
- ○ 30 or over (9)

Q3 Cancer diagnosis

- ◯ Leukaemia (1)
- ◯ Lymphoma (2)
- ◯ Brain or spinal cord tumour (3)
- ◯ Bone or soft tissue sarcoma (e.g., Ewing sarcoma and osteosarcoma) (4)
- ◯ Germ cell tumour (e.g., testicular) (5)
- ◯ Carcinoma (e.g., Bladder cancer, breast cancer, colorectal, lung etc) (6)
- ◯ Other/not sure (7)

Q4 Has your cancer come back at any point during or after treatment?

- ◯ Yes (1)
- ◯ No (2)

Q5 Sex

- ◯ Male (1)
- ◯ Female (2)
- ◯ Prefer not to say (3)

Q5 What is your ethnicity?

- ◯ White (23)
- ◯ Black or African American (24)
- ◯ American Indian or Alaska Native (25)
- ◯ Asian (26)
- ◯ Native Hawaiian or Pacific Islander (27)
- ◯ Other (28)
- ◯ Prefer not to say (29)

Q6 Have you ever taken part in a cancer clinical trial?

- ◯ Yes (1)
- ◯ No (2)
- ◯ Not sure (3)

End of Block: Demographics questions

Start of Block: ACTS-CT and supplementary questions

Q1 I’d get improved cancer treatment if I took part in a cancer study.

- ○ Strongly disagree (36)
- ○ Disagree (37)
- ○ Somewhat disagree (38)
- ○ Neither agree nor disagree (39)
- ○ Somewhat agree (40)
- ○ Agree (41)
- ○ Strongly agree (42)

Q2 People who join cancer studies have a better chance of beating their cancer.

- ○ Strongly disagree (29)
- ○ Disagree (30)
- ○ Somewhat disagree (31)
- ○ Neither agree nor disagree (32)
- ○ Somewhat agree (33)
- ○ Agree (34)
- ○ Strongly agree (35)

Q3 Joining a cancer study would mean I'd receive the best existing cancer treatment.

- Strongly disagree (9)
- Disagree (10)
- Somewhat disagree (11)
- Neither agree nor disagree (12)
- Somewhat agree (13)
- Agree (14)
- Strongly agree (15)

Q4 By joining a cancer study, I would receive better health care.

- Strongly disagree (9)
- Disagree (10)
- Somewhat disagree (11)
- Neither agree nor disagree (12)
- Somewhat agree (13)
- Agree (14)
- Strongly agree (15)

Q5 Taking part in a cancer study is a lot more trouble than just getting the usual treatment.

- Strongly disagree (9)
- Disagree (10)
- Somewhat disagree (11)
- Neither agree nor disagree (12)
- Somewhat agree (13)
- Agree (14)
- Strongly agree (15)

Q6 Getting treatment in a cancer study is less safe than getting the usual treatment.

- Strongly disagree (9)
- Disagree (10)
- Somewhat disagree (11)
- Neither agree nor disagree (12)
- Somewhat agree (13)
- Agree (14)
- Strongly agree (15)

Q7 Treatments received in a cancer study could be unsafe for myself.

- Strongly disagree (9)
- Disagree (10)
- Somewhat disagree (11)
- Neither agree nor disagree (12)
- Somewhat agree (13)
- Agree (14)
- Strongly agree (15)

Q8 My taking part in a cancer study could lead to more health problems.

- Strongly disagree (9)
- Disagree (10)
- Somewhat disagree (11)
- Neither agree nor disagree (12)
- Somewhat agree (13)
- Agree (14)
- Strongly agree (15)

Q9 Joining a cancer study would make cancer treatment more difficult.

- Strongly disagree (9)
- Disagree (10)
- Somewhat disagree (11)
- Neither agree nor disagree (12)
- Somewhat agree (13)
- Agree (14)
- Strongly agree (15)

Q10 In general, people should know more about cancer studies.

- Strongly disagree (9)
- Disagree (10)
- Somewhat disagree (11)
- Neither agree nor disagree (12)
- Somewhat agree (13)
- Agree (14)
- Strongly agree (15)

Q11 Cancer studies are of little importance to me.

- Strongly disagree (9)
- Disagree (10)
- Somewhat disagree (11)
- Neither agree nor disagree (12)
- Somewhat agree (13)
- Agree (14)
- Strongly agree (15)

Q12 Access to cancer treatment studies is important to me.

- Strongly disagree (9)
- Disagree (10)
- Somewhat disagree (11)
- Neither agree nor disagree (12)
- Somewhat agree (13)
- Agree (14)
- Strongly agree (15)

Q13 People who take part in cancer studies are helping all of us fight cancer.

- Strongly disagree (9)
- Disagree (10)
- Somewhat disagree (11)
- Neither agree nor disagree (12)
- Somewhat agree (13)
- Agree (14)
- Strongly agree (15)

Q14 I feel certain my safety would be watched closely in a cancer study.

- Strongly disagree (9)
- Disagree (10)
- Somewhat disagree (11)
- Neither agree nor disagree (12)
- Somewhat agree (13)
- Agree (14)
- Strongly agree (15)

Q15 Doctors and nurses tell patients the truth about what to expect during a cancer study.

- Strongly disagree (9)
- Disagree (10)
- Somewhat disagree (11)
- Neither agree nor disagree (12)
- Somewhat agree (13)
- Agree (14)
- Strongly agree (15)

Q16 If I took part in a cancer study, I would be treated like a guinea pig.

- Strongly disagree (9)
- Disagree (10)
- Somewhat disagree (11)
- Neither agree nor disagree (12)
- Somewhat agree (13)
- Agree (14)
- Strongly agree (15)

Q17 Doctors and nurses mislead their patients who are involved in cancer studies.

- Strongly disagree (9)
- Disagree (10)
- Somewhat disagree (11)
- Neither agree nor disagree (12)
- Somewhat agree (13)
- Agree (14)
- Strongly agree (15)

Q18 It would be safe for me to join a cancer study for treatment

- Strongly disagree (9)
- Disagree (10)
- Somewhat disagree (11)
- Neither agree nor disagree (12)
- Somewhat agree (13)
- Agree (14)
- Strongly agree (15)

Q19 I would be more likely to participate in a clinical trial if my current cancer treatment was not effective or available.

- Strongly disagree (9)
- Disagree (10)
- Somewhat disagree (11)
- Neither agree nor disagree (12)
- Somewhat agree (13)
- Agree (14)
- Strongly agree (15)

Q20 The greater the severity of my illness/cancer, the more likely I would be willing to participate in a clinical trial.

- Strongly disagree (9)
- Disagree (10)
- Somewhat disagree (11)
- Neither agree nor disagree (12)
- Somewhat agree (13)
- Agree (14)
- Strongly agree (15)

Q21 It doesn’t matter who approaches me about a clinical trial as long as they have all of the necessary information to answer any questions that I have.

- ◯ Strongly disagree (9)
- ◯ Disagree (10)
- ◯ Somewhat disagree (11)
- ◯ Neither agree nor disagree (12)
- ◯ Somewhat agree (13)
- ◯ Agree (14)
- ◯ Strongly agree (15)

Q22 I am worried about how a clinical trial can affect my daily life or short-term goals (work and school).

- ◯ Strongly disagree (9)
- ◯ Disagree (10)
- ◯ Somewhat disagree (11)
- ◯ Neither agree nor disagree (12)
- ◯ Somewhat agree (13)
- ◯ Agree (14)
- ◯ Strongly agree (15)

Q23 I am worried about how a clinical trial can affect my long-term goals (starting a family, career/other goals).

- Strongly disagree (9)
- Disagree (10)
- Somewhat disagree (11)
- Neither agree nor disagree (12)
- Somewhat agree (13)
- Agree (14)
- Strongly agree (15)

Q24 After being approached for a clinical trial, I would seek additional information from the Internet.

- Strongly disagree (9)
- Disagree (10)
- Somewhat disagree (11)
- Neither agree nor disagree (12)
- Somewhat agree (13)
- Agree (14)
- Strongly agree (15)

Q25 My friends’ and family’s opinions matter to me when making a decision about clinical trials.

- Strongly disagree (9)
- Disagree (10)
- Somewhat disagree (11)
- Neither agree nor disagree (12)
- Somewhat agree (13)
- Agree (14)
- Strongly agree (15)

Q26 I am too overwhelmed to consider participation in a clinical trial.

- Strongly disagree (9)
- Disagree (10)
- Somewhat disagree (11)
- Neither agree nor disagree (12)
- Somewhat agree (13)
- Agree (14)
- Strongly agree (15)

Q27 I would consider my ability to meet the demands of the trial protocol (ie, frequency of visits, procedures, travel time).

- Strongly disagree (9)
- Disagree (10)
- Somewhat disagree (11)
- Neither agree nor disagree (12)
- Somewhat agree (13)
- Agree (14)
- Strongly agree (15)

End of Block: ACTS-CT and supplementary questions
